# The gut microbiota's role in bulimia nervosa and binge eating disorder: etiological insights and therapeutic implications from a scoping review

**DOI:** 10.1016/j.nsa.2025.105526

**Published:** 2025-08-19

**Authors:** Silvia Tempia Valenta, Anna Rita Atti, Federica Marcolini, Daniele Rossi Grauenfels, Gaia Giovannardi, Giuseppe Fanelli, Diana De Ronchi

**Affiliations:** a Department of Biomedical and Neuromotor Sciences, University of Bologna, Bologna, Italy; b Research Group on Emergency and Disaster Medicine, Vrije Universiteit Brussel, Brussels, Belgium; c Department of Human Genetics, Radboud University Medical Center, Donders Institute for Brain, Cognition and Behaviour, Nijmegen, the Netherlands

**Keywords:** Gut microbiota, Gut bacteria, Gut-brain-axis, Compulsive behavior, Overeating behavior, Cross-reactivity

## Abstract

This scoping review synthesizes emerging evidence on the relationship between gut microbiota and eating disorders (EDs), particularly bulimia nervosa (BN) and binge eating disorder (BED). An electronic search was conducted in the PubMed/MEDLINE and Web of Science databases, spanning from their inception until December 2023. From an initial pool of 166 records, 14 articles were included and qualitatively synthesized. Two overarching themes emerged. The first focuses on etiological hypotheses, pointing to autoimmunity and psychoneuroendocrinoimmunological mechanisms as potential mediators in the association between ED pathophysiology and the gut microbiota. Significant findings include the identification of elevated levels of bacterial caseinolytic protease B protein in patients, correlating with symptom severity and specific autoantibodies. Furthermore, studies indicate that gut microbiota proteins might trigger autoantibody production. Distinct microbial profiles are associated with different ED subtypes and behaviors. The second theme examines the clinical implications of this link. Human trials demonstrate the potential of probiotic interventions in improving eating-related clinical outcomes post-bariatric surgery. Murine models also demonstrate the role of gut microbiota in controlling compulsive overeating, with probiotic interventions proving effective in reducing binge eating and anxiety-like behaviors. In conclusion, this review summarizes current research and highlights the interactions among the gut microbiota, immune system, and hormonal regulation in the development and progression of BN and BED. It also highlights the potential for ongoing interdisciplinary research to identify possible underlying mechanisms and reliable biomarkers, with the ultimate goal of developing targeted therapeutic interventions for BN and BED.

## 1 Introduction

In recent years, growing research has investigated the relationship between gut microbiota and different health conditions, ranging from metabolic diseases, such as diabetes mellitus and obesity, to psychiatric manifestations, including anxiety, mood and neurodevelopmental disorders (Lloyd-Price et al., 2016; Seitz et al., 2019; Sukmajaya et al., 2021).

The gut microbiota (the community of microorganisms) and its microbiome (their collective genomes and functions) exert a substantial influence on essential physiological processes contributing to overall well-being (Seitz et al., 2019). Indeed, it is actively involved in nutrient fermentation, a process essential for breaking down and absorbing vital substances, and it significantly impacts metabolic functions and body-weight regulation (Seitz et al., 2019). Additionally, the gut microbiota influences hormonal balance, consequently affecting various endocrine functions related to appetite, stress response, and systemic homeostasis (Slyepchenko et al., 2016). Its role extends to maintaining the integrity of the gut barrier, influencing gut permeability and, accordingly, inflammatory responses (Dickson, 2016). Notably, the gut microbiota's influence is not confined to physical processes alone; it extends to behavioral patterns and mental health through the bidirectional network known as the gut-brain axis (Gilbert et al., 2018). The functionality of this interconnected network is dependent on the homeostatic balance of the gut microbiota, which regulates physiological processes ranging from cellular function to cognitive health (Nguyen et al., 2018).

A particularly interesting area of study is the potential role of the gut microbiota in eating disorders (EDs) (Ghenciulescu et al., 2021; Smitka et al., 2021). Much of the current research has centered on anorexia nervosa (AN), yielding outcomes that continue to be a subject of debate. Studies examining microbiota diversity, both in terms of species richness (alpha diversity) and composition (beta diversity), as well as the relative abundance of taxa in individuals with AN, have produced findings that still remain contentious (Slyepchenko et al., 2016; Guinane et al., 2013; van de Wouw et al., 2017; Herpertz-Dahlmann et al., 2017; Di Lodovico et al., 2021; Garcia et al., 2023). One possible explanation for these discrepancies is the impact of refeeding practices, which are known to directly influence microbial composition (Kleiman et al., 2015; Butler et al., 2021). In addition, several mechanisms have been proposed to explain how the gut microbiota may be linked to AN, including increased intestinal permeability, low-grade systemic inflammation, the presence of autoantibodies, and impaired neurogenesis (Fetissov et al., 2002, 2008; Ruusunen et al., 2019).

Bulimia nervosa (BN) and binge eating disorder (BED), characterized by episodes of excessive food consumption within short time frames, are other prevalent and disabling EDs (Fairburn et al., 2000). Although they are increasingly recognized and diagnosed, the multifactorial etiology of BN and BED remains enigmatic, with a blend of psychosocial, genetic, and biological factors contributing to their development and perpetuation (Kendler et al., 1992; Fairburn et al., 1997, 1998; Yao et al., 2021; Donato et al., 2022). While the psychological and social determinants have been extensively described (Brambilla, 2001; Solmi et al., 2020; Burton et al., 2019), the involvement of biological factors, which include but are not limited to neurohormones regulating hunger and satiety, as well as neurotransmitters governing mood and anxiety, contribute to an additional layer to our understanding of these disorders (Brambilla, 2001; Mathes et al., 2009). Among biological factors, some recent studies have indicated a relationship between gut microbiota composition and the regulation of appetite, mood, and body mass in patients suffering from BN and BED (Herman and Bajaka, 2021). However, our understanding of the possible role of gut microbiota in BN and BED is still in its nascent stages.

In light of this, our scoping review aims to map the current landscape of research and identify main themes and gaps in the literature concerning the gut microbiota's possible relationship with BN and BED.

## 2 Materials and methods

This scoping review was conducted in accordance with the Preferred Reporting Items for Systematic Reviews and Meta-Analyses Extension for Scoping Reviews (PRISMA-ScR) guidelines (Tricco et al., 2018). A narrative data synthesis method was used to derive macro-themes inductively by synthesizing data from the relevant literature. Considering the nature of this review, which summarizes data from previously published studies, ethical approval was not deemed necessary. The review process was streamlined using the Rayyan program, an AI-powered tool designed for systematic literature reviews (Ouzzani et al., 2016). This tool was instrumental in expediting the initial screening and eliminating duplicate entries.

### 2.1 Search strategy

Two authors (D.R.G. and G.G.) independently searched the scientific literature across PubMed/MEDLINE and Web of Science databases, covering the period from inception until December 10th, 2023. In instances where discrepancies arose during the study selection phase, resolution was achieved through open discussions among the involved parties. In the event of persistent disagreement, a third supervisor (S.T.V.) was consulted to provide additional insights and contribute to the decision-making process.

The search query was developed to encompass keywords relevant to the two primary concepts: BN/BED and gut microbiota. The full search queries are available in the **Supplementary Materials**. Given the paucity of previous literature on this subject, keywords were searched in the titles, abstracts, and full texts to maximize the likelihood of retrieving relevant articles. Beyond keyword searching, we also identified studies through alternative sources. Citation chaining was employed during full-text screening to ensure the inclusion of relevant literature that might have been overlooked in the initial searches.

### 2.2 Eligibility criteria

We included articles (1) addressing the relationship between dysregulated eating behaviors, such as those observed in BN, BED, and relevant animal models, and the gut microbiota, (2) written in English. We exclude articles that (1) focused on non-target populations (e.g., individuals with overweight or obesity, healthy subjects, murine models not relevant to BN or BED), (2) addressed non-target topics (e.g., studies on ED symptoms or treatments), (3) non-target design (i.e., review articles).

The inclusion and exclusion criteria were initially applied to screen titles and abstracts. Following this screening, articles meeting these criteria underwent a full-text examination to evaluate their relevance to this review. In the screening and evaluation phase, both reviewers (D.R.G. and G.G.) actively engaged in assessing the match of articles with the inclusion and exclusion criteria. In instances of discrepancies or uncertainties, a third reviewer (S.T.V.) was consulted to provide additional insights and contribute to the decision-making process.

### 2.3 Data extraction and management

Two authors independently extracted data from the eligible articles following this scheme: reference, study design, sample characteristics, main objective(s), interventions, measurement, and main findings. A third reviewer resolved any discrepancies.

## 3 Results

**Fig. 1:**
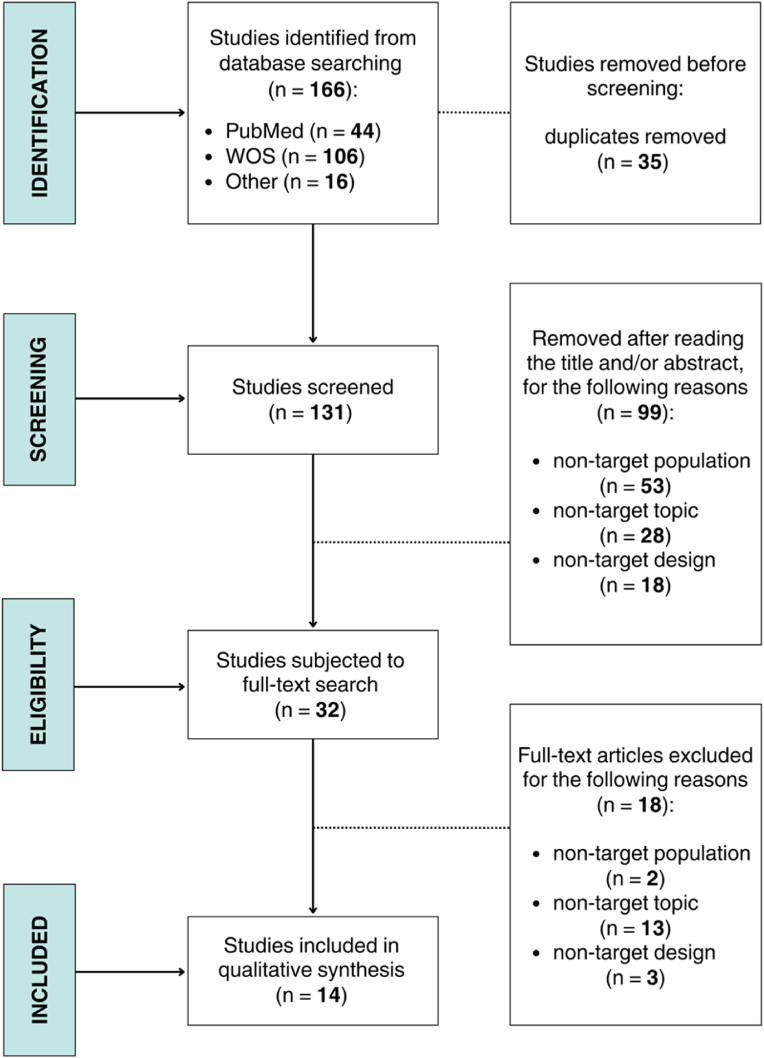
Flowchart of study selection.

A flowchart summarizing the different phases of the selection process is depicted in Fig. 1.

From the initial 166 records, 14 articles were selected for inclusion. Table 1 presents a summary of main characteristics extracted from the included articles, arranged in the same order as they are presented in the results section. Although most studies have addressed different EDs including AN, each included study individually examined BN and/or BED, allowing for the extraction of specific information. The studies spanned various continents, with France emerging as a prominent contributor with 5 studies out of 16 (Tennoune et al., 2014, 2015; Breton et al., 2016; Galmiche et al., 2020; Lengvenyte et al., 2022). The sample size ranged from 20 to 7960. The age range of human participants varied, with a mean age ranging from 17 to 50 years.

**Table 1:** The main characteristics of the included articles.

Reference	Study design (Subjects)	Sample characteristics	Main objective(s)	Interventions	Measurement	Main findings
Theme 1: Etiological hypothesis						
*Autoimmunity and psychoneuroendocrinoimmunological models*						
Tennoune et al., 2014 (France)	Experimental (mice) and cross- sectional (humans)	AN, BN, and BED **SS** 73 (32 with BN, 14 with BED); 73 F, 0 M **MA (YO)** BN = 22.7 ± 6.8 BED = 30.6 ± 11.6 Mice: Two-Month-old male C57Bl6 mice	• The aim of the present study was to determine the putative microbial origin of α-MSH auto-Abs and to validate their relevance to EDs	–	Plasma levels of auto-Abs reacting with ClpB, α-MSH, and adrenocorticotropic hormone were measured using enzyme-linked immunosorbent assay	• Bacterial ClpB protein, produced by several intestinal microorganisms, can be responsible for the production of auto-Abs crossreactive with α-MSH • α-MSH crossreactive anti-ClpB IgGs were increased in all of three groups patients with ED, in particular BN and BED
Breton et al., 2016 (France)	Cross- sectional (humans)	AN, BN, and BED **SS** 95 (29 with BN and 13 with BED); 95 F, 0 M **MA (YO)** BN = 21.6 ± 1.2 BED = 27 ± 2.9	• Since bacterial ClpB is naturally present in human plasma, this study wanted to verify its presence in EDs	–	Measurement of plasma concentration of ClpB	• Bacterial ClpB concentrations are elevated in EDs and correlate with the EDI-2 scores, α-MSH, anti-ClpB Abs, and anti-α-MSH Abs • These results support a link between bacterial ClpB and the ED pathophysiology
Rantala et al., 2019 (Finland)	Qualitative (humans)	AN, BN, and BED **SS** - **MA (YO)** -	• This article reviewed ultimate-level hypotheses regarding the origins of EDs, and synthesized them with a novel proximate explanation	–	–	• An evolutionary psychoneuroimmunological model is proposed to explain EDs not as separate diseases but as a continuum • It includes the gut microbiota as a key factor
Galmiche et al., 2020 (France)	Qualitative and cross- sectional (humans)	AN, BN, and BED **SS** 120 (12 with BN, 67 with BED); 103 F, 17 M **MA (YO)** BN = 36.0 ± 16.5 BED = 39.4 ± 12.3	• This study wanted to measure different peptides and Ig resulting from dysbiosis and altered microbiota in EDs	A few patients in each group received some non‐specific symptomatic treatments	–	• Dysbiosis and microbiota-derived signals can overstimulate endocrine cells, producing orexigenic and anorexic peptides • Changes in the concentration and/or affinity of several anti-peptide IgG may contribute to the physiopathology of EDs
Terry et al., 2022 (Canada)	Qualitative (humans)	AN and BN **SS** - **MA (YO)** -	• This article made a critical analysis of the bidirectional relationship between gut microbes and biological processes implicated in EDs	–	–	• The gut microbiota, which can be different in individuals with EDs, may be intimately intertwined with the HPA axis, interacting with neurotransmitters as well as with hunger hormones • The impact on hunger and satiety can be elucidated through interactions with the immune system
*Gut microbiota composition*						
Leyrolle et al., 2021 (Belgium)	Cross- sectional (humans)	BED **SS** 101 (42 patients with BED) **MA (YO)** BED = 50.5 ± 10.4	• This study aimed to examine the complex biological and psychiatric profile of individuals with obesity with and without BED • It also wanted to conduct an analysis of the composition of the intestinal microbiota and its evaluation from a metabolomic perspective	–	Stool samples were collected, and Genomic DNA was extracted	• BED subjects show a decrease in genus *Akkermansia* and *Intestinimonas* as well an increase in genus Bifidobacterium and genus *Anaerostipes* • The study shows a tendency of increased beneficial bacteria in patients with BED • These changes in the microbiota seem not to be driven by dietary habits
Fan et al., 2023 (China)	Experimental and cross- sectional (humans and mice)	Humans: BN **SS** 20 (11 with BN); 20 F, 0 M **MA (YO)** 17.45 Mice: overeating model of female mice	• This study focused on the changes in the intestinal microbiota in a mouse model and patients with EDs • It analyzed whether such changes affect related brain regions through the gut-brain axis	–	16S-ribosome DNA sequencing of the gut microbiota	• A loss of genus *Faecalibacterium* is found in patients with BN • A significantly lower KYNA (microbiome-derived metabolite) level is found in patients with BN
Castellini et al., 2023 (Italy)	Cross- sectional (humans)	AN, BN, and BED **SS** 75 (17 with BN and 9 with BED) **MA (YO)** 24.14 ± 7.09	• This study attempted to propose an integrated explicative model for psychopathology associated with EDs • It also wanted to include the presence of childhood adverse experiences, gut microbiota composition, and its metabolites (SCFAs)	10 patients were receiving antidepressant therapy at the time of assessment, while 4 were taking anxiolytics	The study characterized the bacterial fecal microbiota by means of high-throughput sequencing of the V3-V4 region of the 16S rDNA gene. The study quantified the bacterial richness within each sample (alpha-diversity)	• Gut microbiota composition differs between subjects with EDs and healthy controls • The alpha and beta diversity of the microbiota is related to the eating pattern (binge or restriction), and SCFAs levels are linked with psychopathological features • Patients with binge-purging symptoms are characterized by the genus *Prevotella*, while patients with restriction symptoms by the genus *Bacteroides*, genus *Facklamia*, and genus *Lachnospira* • A model proposed is that childhood trauma causes gut dysbiosis
Raevuori et al., 2016 (Finland)	Case-control (humans)	AN, BN, and BED **SS** 7960 (747 with BN, 144 with BED); 7611 F, 340 M **MA (YO)** BN = 25.9 BED = 38.0	• This study examined the use of antimicrobial medications (i.e., antibacterials, antifungals and antivirals) as a proxy for infections in patients with EDs	Prescription data of any antimicrobial medication was collected in a 10-year period	–	• Elevated use of antimicrobial medication in BN, BED and in male with AN is found • A model suggested is that EDs are associated with the dysregulation of microbiota that can potentially lead to infections
Igudesman et al., 2023 (USA)	Cross- sectional (humans)	BN and BED **SS** 265 (147 with BN, 118 with BED) **MA (YO)** 29.0	• This study assessed the association of the composition and diversity of the intestinal microbiota with binge eating and compensatory behaviors	–	16S rRNA gene sequencing of the gut microbiome	• In BN and BED, laxative use reduced gut microbial diversity • In particular, this includes a reduction in the beneficial genus *Alistipes* and species *Eubacterium ventriosum*, as well as in the pathobionts genus *Bilophila* and genus GCA900066575 of the *Firmicutes* phylum
Lengvenyte et al., 2022 (France)	Cross- sectional (humans)	AN and BN **SS** 277 (97 with BN); 277 F, 0 M **MA (YO)** 27.65 ± 10.17	• This study aimed to investigate whether the misuse of laxatives was associated with suicide attempts in patients affected by AN or BN	–	–	• Laxative misuse is associated with suicidal attempts and ideation in patients with ED • This can be due to a perturbation in the gut microbiota, as laxative use is known to increase the relative number of genus *Bacteroides* and genus *Alistipes*
***Theme 2: Clinical implications***						
*From studies on humans*						
Carlos et al., 2022 (Brazil)	RCT	FA and BED **SS** 101 (15 patients with BED) **MA (YO)** 40 ± 11.25	• This study aimed to assess the effect of probiotic supplementation on BED and FA in subjects after Roux-en-Y gastric bypass surgery	The supplements given to the patients were Lactobacillus and Bifidobacterium	–	• In comparison to the placebo group, the probiotic recipients demonstrated significantly lower levels of FA symptoms and reduced scores for binge eating • This suggests a potential long-term benefit of probiotic intervention in mitigating FA and binge eating outcomes following bariatric surgery
*From studies on murine models*						
Tennoune et al., 2015 (France)	Experimental- preclinical (rats)	48 adult Wistar rats	• This study aimed to compare the effects of *Escherichia coli* on feeding and auto-Abs against α-MSH and adrenocorticotropic hormone (ACTH), between female and male rats	–	*Escherichia coli* DNA was purified from the feces of rats and extracted from the cultures of the strain	• Species *E. coli* provision is accompanied by an increase in body weight gain in females but a decrease in body weight gain and food intake in males • Females respond to *E. coli* by increasing α-MSH IgG levels and affinity, males by increasing α-MSH IgM levels • The affinity of IgG for ACTH was increased in both Escherichia coli-treated females and males, although with different kinetics • As a consequence, it is suggested that sex-related presence of *E. coli* may represent a risk factor for ED development
Agustí et al., 2021 (Spain)	Experimental- preclinical (rats)	45 validated rat model of FA	• This study sought to evaluate the action of the gut microbiota in a validated rat model of FA, where loss of dietary intake control was induced by intermittent periods of fasting	Rats were divided into three experimental groups: a control group, an IF group, and an IF group treated with Bacteroides uniformis	–	• The administration of species *Bacteroides uniformis* in a rat FA model ameliorates binge eating and decreases anxiety-like behavior • *Bacteroides uniformis* reverses the fasting-induced microbiota changes and increases the abundance of species linked to healthy metabolic types
Fan et al., 2023 (China)	Experimental and cross- sectional (humans and mice)	373 overeating model of female mice	• This study focused on the changes in the intestinal microbiota in a mouse model • It analyzed whether alterations in intestinal microbiota are responsible for excessive intake of palatable foods	–	16S rRNA gene sequencing of the gut microbiome	• A loss of genus *Faecalibacterium* and a significantly lower KYNA level is found • The transplantation of a probiotic (species *Faecalibacterium prausnitzii*) or dietary supplement of KYNA alleviates OD symptoms

***Legend.*** Abs, antibodies; AG, acyl ghrelin; AN, anorexia nervosa; BED, binge eating disorder; BMI, Body Mass Index; BN, bulimia nervosa; ClpB, Caseinolytics protease B; DAG, des-acyl ghrelin; *E. coli*, *Escherichia coli*; ED, eating disorder; EDI-2, ED Inventory-2; F, female; FA, food addiction; GLP-1, glucagon-like peptide 1; HPA, hypothalamic-pituitary-adrenal; IF, intermittent fasting; Ig, immunoglobulin; M, male; MA, mean age; KYNA, kynurenic acid; OD, overeating disorder; PYY, peptide YY; SCFA, short chain fatty acid; SPF, specific pathogen-free; SS, sample size; YO, years old: α-MSH, α melanocyte-stimulating hormone; -, not reported or not applicable.

**Fig. 2:**
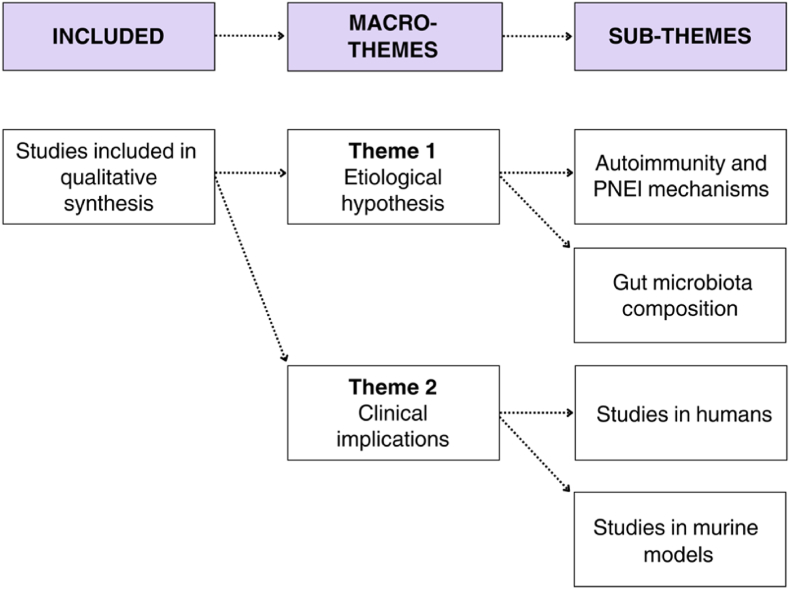
Macro-themes and respective sub-themes ***Legend.*** PNEI, psychoneuroendocrinoimmunological.

We identified two overarching macro-themes, illustrated in Fig. 2. The (1) first macro-theme concerns the etiological hypotheses linking gut microbiota and BN/BED, encompassing the two sub-themes of (1a) autoimmunity and psychoneuroendocrinoimmunological (PNEI) mechanisms and (1b) the composition of the gut microbiota. The (2) second macro-theme focuses on the clinical implications of the relationship between BN/BED and the gut microbiota as revealed by studies in (2a) humans and (2b) murine models.

### 3.1 Macro-theme 1: Etiological hypotheses

#### 3.1.1 Autoimmunity and psychoneuroendocrinoimmunological (PNEI) mechanisms

Five studies have proposed models suggesting that autoimmunity and PNEI mechanisms may underlie the etiology of BN and BED. These studies were either qualitative or cross-sectional, including both murine and human models.

In particular, research showed that gut bacterial caseinolytic protease B (ClpB), produced by several intestinal microorganisms, is implicated in the production of autoantibodies cross-reactive with α-melanocyte-stimulating hormone (α-MSH), which are associated with altered feeding and emotional behaviors in patients with EDs, in particular those with BN and BED (Tennoune et al., 2014). Another study consistently identified elevated gut bacterial ClpB protein levels in patients with EDs (Breton et al., 2016). ClpB levels correlated with ED symptom severity, anti-ClpB antibodies, and anti-α-MSH antibodies, supporting a potential link between bacterial ClpB and the pathophysiology of EDs (Breton et al., 2016).

A new perspective emerges with the proposition of an evolutionary psychoneuroimmunological (PNEI) model of EDs (Rantala et al., 2019). According to this theory, the combination of modern food environments, rich in highly caloric foods, and evolutionary mating instincts may lead to chronic stress and contribute to the development of disordered eating behaviors (Rantala et al., 2019). This ongoing stress is believed to trigger both neuroinflammation and gut dysbiosis (Rantala et al., 2019). Supporting this idea, other studies have found that gut microbiota imbalances can overstimulate endocrine cells, leading to abnormal production of hormones regulating hunger and fullness (i.e., orexigenic and anorexic peptides) (Galmiche et al., 2020). These changes may also affect the immune system. Specifically, variations in the concentration and/or affinity of various anti-peptide immunoglobulin G (IgG) antibodies-those that interact with these peptides-might influence the development of EDs. In fact, resistance to these peptides, which has been observed in individuals with EDs, could be linked to how the body produces or responds to these antibodies (Galmiche et al., 2020).

This perspective also extends to the endocrine system, highlighting how the gut microbiota may interact with key components such as the hypothalamic-pituitary-adrenal (HPA) axis, neurotransmitters, and hormones (Terry et al., 2022). These interactions can influence how we experience hunger and fullness, often through their effects on the immune system (Terry et al., 2022). For instance, people with BN tend to have lower levels of cortisol in their blood, which can reduce feelings of fullness and potentially contribute to binge-eating episodes (Terry et al., 2022). Early life stress might play a role in this, as it can influence the development of the HPA axis and influence how hunger and satiety signals are regulated later in life (Terry et al., 2022).

#### 3.1.2 Gut microbiota composition

Six cross-sectional studies have examined the gut microbiota composition in individuals with BN and BED, consistently demonstrating specific microbial profiles associated with different eating behaviors or medication use.

Recent research shows notable differences in both the types and amounts of distinct gut bacteria and metabolites in these disorders (Leyrolle et al., 2021). Individuals with BED were found to have reduced levels of genus *Akkermansia* and genus *Intestinimonas* alongside increased levels of genus *Bifidobacterium* and genus *Anaerostipes*, when compared to individuals with obesity who did not have BED (Leyrolle et al., 2021). Notably, these differences persisted regardless of dietary habits (Leyrolle et al., 2021).

In the case of BN, a study reported a reduction in genus *Faecalibacterium* and in the microbiome-derived metabolite kynurenic acid (KYNA) levels (Fan et al., 2023). Another recent study comparing individuals with EDs to healthy controls found that the diversity of gut microbiota, both in alpha and beta diversity, was linked to specific ED symptoms. The researchers identified the following distinctive microbiota profiles: those with binge-purge behaviors had higher levels of genus *Prevotella*, while those with restrictive eating patterns had more genus *Bacteroides*, genus *Facklamia*, genus *Lachnospira* (Castellini et al., 2023). Notably, this study also found a connection between childhood trauma and changes in gut microbiota among people with EDs (Castellini et al., 2023).

Other research also considered possible iatrogenic influences on gut microbiota composition. Individuals with BN and BED were found to use more antimicrobial drugs, including antibiotics, antifungals, and antivirals, than those without EDs (Raevuori et al., 2016). This raises the possibility that EDs may either lead to increased use of antimicrobial medication (e.g., EDs associated with microbiota dysregulation leading to infections) or be a consequence of escalated antimicrobial medication use (Raevuori et al., 2016). Two recent studies have also explored the effects of laxative use, which is common among patients with EDs, on gut microbiota composition. One study linked laxative misuse in patients with BN to suicidal attempts and ideation, potentially due to gut microbiota perturbations (e.g., increased genus *Bacteroides* and genus *Alistipes*) (Lengvenyte et al., 2022). Another study reported that laxative use was associated with reduced microbial diversity in patients with BN and BED (i.e., Shannon diversity, Faith phylogenetic diversity, and Peilou's evenness). This included decreases in beneficial genus *Alistipes* and species *Eubacterium ventriosum*, as well as in the pathobionts genus *Bilophila* and genus GCA900066575 of the *Firmicutes phylum* (Igudesman et al., 2023).

### 3.2 Macro-theme 2: Clinical implications

#### 3.2.1 Studies in humans

A randomized controlled trial on individuals with food addiction and BED undergoing Roux-en-Y gastric bypass surgery revealed that a three-month probiotic supplementation could have long-term benefits in improving food addiction and binge eating outcomes (Carlos et al., 2022). In particular, the probiotic recipients demonstrated significantly lower levels of food addiction symptoms and reduced scores for binge eating compared to the placebo group (Carlos et al., 2022).

#### 3.2.2 Studies in murine models

Three experimental studies on murine models showed the potential of microbiota-based interventions for controlling compulsive overeating behaviors. A research utilizing a rat model explored sex-related differences in ED development, focusing on the influence of *Escherichia coli (E. coli)* supplementation on feeding behavior and autoantibodies against α-MSH and adrenocorticotropic hormone (ACTH) (Tennoune et al., 2015). Over a 3-week period, female rats showed increased body weight gain with *E. coli* supplementation, while males experienced decreased weight gain and food intake (Tennoune et al., 2015). Both sexes exhibited elevated plasma levels of anti-α-MSH and -ACTH IgG, with females responding to *E. coli* by enhancing α-MSH IgG levels and affinity, and males by increasing α-MSH IgM levels (Tennoune et al., 2015).

A study utilizing a rat model to simulate food addiction found that the administration of species *Bacteroides uniformis* ameliorated binge eating behavior and reduced anxiety-like symptoms (Agustí et al., 2021). Similarly, a recent study employing an overeating mouse model to mirror human BN showed the effectiveness of different interventions, including the administration of a probiotic (specifically, *Faecalibacterium prausnitzii*) and supplementation with KYNA, in the view of addressing its deficiency in individuals with BN (Fan et al., 2023).

## 4 Discussion

The present review summarized evidence from both human and animal studies highlighting a bidirectional relationship between dysregulated eating behaviors, such as those seen in BN and BED, and the gut microbiota. A growing number of studies suggest that autoimmune and PNEI mechanisms, including the production of antibodies against microbial proteins like ClpB, may be potential mediators in the pathophysiology of EDs. Alterations in the gut microbiota composition have also been consistently observed, with distinct bacterial signatures linked to different eating behaviors, symptom severity and even early life stress. Importantly, changes at the microbiome level (functional outputs such as KYNA) highlight the metabolic contribution of microbial genes and pathways. Interestingly, both pharmacological and behavioral factors, such as antimicrobial and laxative use, appear to further influence microbial profiles in BN and BED. In terms of clinical implications, these findings are beginning to inform new treatment directions. Probiotic supplementation, for instance, shows promise in reducing food addiction symptoms in humans following bariatric surgery, while preclinical studies in rodents have demonstrated that specific microbial interventions can modulate binge eating, anxiety-like behavior, and immune responses.

Initial evidence linking EDs with autoimmunity dates back to the late 1990s. The pioneering studies by Corcos et al. (1999) identified autoantibodies reactive to dopamine and serotonin in individuals with BN, laying the foundation for subsequent investigations (Corcos et al., 1999). Later research revealed autoantibodies targeting α-MSH, adrenocorticotropic hormone (ACTH), and luteinizing hormone-releasing hormone (LHRH) in subpopulations of patients with different EDs, including BN and BED (Fetissov et al., 2002), proposing the hypothesis that there was an involvement of autoantibodies against neuropeptides in the regulation of appetite and emotions in EDs (Fetissov et al., 2008). Fetissov and Hökfelt (2019) advanced this concept by proposing a model centered on ClpB, a bacterial antigen-specific mimetic of α-MSH, which triggers the production of autoantibodies cross-reactive to α-MSH. These autoantibodies would form immune complexes with α-MSH, chronically activating the melanocortin system, an important player in the regulation of feeding behavior (Fetissov et al., 2019).

Epidemiological data from large cohorts further reinforced the autoimmune hypothesis. Raevuory et al. (2014) and Hedman et al. (2019) confirmed a significant association between EDs and autoimmune diseases (Raevuori et al., 2014; Hedman et al., 2019). In particular, Raevuory's study revealed a significantly higher prevalence of autoimmune diseases (8.9 %) in individuals with EDs than in the general population (5.4 %) (Raevuori et al., 2014). The diseases in question were mainly those affecting the endocrine and gastrointestinal systems, such as type 1 diabetes mellitus and Crohn's disease (Raevuori et al., 2014). Similarly, Hedman's nationwide survey conducted in Sweden, including more than 2.5 million individuals, showed a strong bidirectional relationship between EDs and autoimmune diseases (Hedman et al., 2019). Again, this link was highlighted particularly with regard to conditions related to the gastrointestinal tract such as celiac disease and Crohn's disease (Hedman et al., 2019).

The PNEI model proposed by Rantala et al. (2019) offers a summary that a reciprocal interaction between chronic stress, neuroinflammation, gut microbiota would contribute to the genesis of EDs (Rantala et al., 2019). Anticipating this line, previous studies had also shown general immunological alterations in BN, with proliferative lymphocyte responses and lower leptin levels (Nagata and Yamada, 2006). In addition, alterations in food-regulating substances such as ghrelin and endocannabinoids have been found in both BN and AN (Monteleone et al., 2011). Research has also shown disturbances in neuroendocrine signaling of hunger and satiety, along with interactions with the mesolimbic dopamine system, which would contribute to core ED symptoms (Berner et al., 2019). A recent study shows that, while AN is associated with increased proinflammatory cytokines, BN exhibit reduced anti-inflammatory cytokine activity (Butler et al., 2021).

Regarding microbiota composition, our study found distinct patterns associated with bulimic and binge-type eating behaviors. In BN, reductions in the abundance of *Faecalibacterium* and lower levels of KYNA (i.e., a microbial metabolite with neuroprotective properties) have been observed (Fan et al., 2023). BED, on the other hand, is characterized by decreased levels of *Akkermansia* and *Intestinimonas*, along with increased levels of *Bifidobacterium* and *Anaerostipes*, changes that appear to be independent of dietary intake (Leyrolle et al., 2021). Both disorders also display altered microbial diversity linked to specific behavioral patterns: binge-purging is associated with an increased abundance of *Prevotella*, whereas restrictive behaviors are linked to higher levels of *Bacteroides*, *Facklamia*, and *Lachnospira* (Castellini et al., 2023). Conversely, existing studies on AN reveal a distinctly different microbiota profile, with globally reduced bacterial load and diminished abundance of certain key taxa, suggesting a compromised metabolic capacity (Armougom et al., 2009; Morita et al., 2015; Carr et al., 2016; Mack et al., 2016; Bulik et al., 2021). While some overlap exists in microbial taxa, such as the presence of *Bacteroides*, *Facklamia*, and *Lachnospira* in restrictive eating patterns, the broader composition and function of the gut microbiota in AN diverge markedly from those in BN and BED (Castellini et al., 2023). Moreover, AN is uniquely characterized by disruptions in the gut virome, particularly a reduction in viral-bacterial interactions. Although elevated ClpB levels, implicated in autoimmune signaling, are found in both AN and BN, the immune response appears to differ: BN is associated with mechanisms enhancing hunger, while AN is more aligned with pathways that promote satiety and suppress intake (Fan et al., 2023). Metabolomic profiles in AN further underscore this distinction, with increased levels of molecules such as indole-3-propionic acid, linked to reduced food consumption, highlighting a metabolic environment adapted to starvation (Mallorquí-Bagué et al., 2018).

According to the view that EDs are a continuum, as suggested by Rantala et al. (2019) and many studies concerning various declinations of these disorders (Mallorquí-Bagué et al., 2018; Munguía et al., 2021), we can think of the gut microbiota as playing a role in directing nonspecific symptoms toward diagnosis-specific constructs. When the underlying mechanisms are examined, clear physiological differences emerge: AN is typically associated with increased insulin sensitivity, whereas BN and BED tend to show reduced insulin sensitivity (Ilyas et al., 2019). This view also takes into account a number of influencing factors such as age (Hopkins et al., 2002), eating patterns, and diet composition (Klingbeil et al., 2018), as well as compensatory behaviors like eliminatory conducts and exercise (Balci et al., 2022; Mach et al., 2017). The use of medications can play an additional role: substances such as laxatives and antidepressants, the use of which is common in this clinical population, have been shown to affect the microbiota composition (Lengvenyte et al., 2022; Raevuori et al., 2016; Igudesman et al., 2023; Borgiani et al., 2025).

When it comes to the treatment of EDs, the current gold standard is early multidisciplinary care involving coordinated psychological and nutritional interventions (Monteleone et al., 2022). However, there are still major institutional and psychological barriers that prevent many people from accessing these services (Tempia Valenta et al., 2024a, 2025; Acle et al., 2021). On top of that, despite how serious and complex these conditions are, there are still very few medications that are officially approved or reliably effective (Monteleone et al., 2022; Marcolini et al., 2024). To date, fluoxetine is the only FDA-approved medication specifically indicated for the treatment of BN at a dosage of 60 mg (Commissioner), while other pharmacological options, such as glucagon-like peptide-1 (GLP-1) receptor agonists, are still under investigation (Tempia Valenta et al., 2023, 2024b; Chen et al., 2024). Given this therapeutic gap, recognizing the role of the body, particularly gut health, as a modifiable factor opens promising new avenues for intervention. Recent proposals include the development of monoclonal antibodies derived from circulating autoantibodies that target appetite-regulating neuropeptides and neurotransmitters, an approach being explored for both AN and BN (Smitka et al., 2021; Hofbauer et al., 2008). In line with this, our findings support the hypothesis that microbiota-based interventions could be effective in managing BN and BED. In one trial, individuals with BED who received probiotic supplementation following gastric bypass surgery showed significant reductions in both food addiction symptoms and binge eating scores compared to a placebo group (Carlos et al., 2022). Murine studies further reinforce these findings, showing that specific microbial treatments can modulate compulsive overeating behaviors (Tennoune et al., 2015; Fan et al., 2023; Agustí et al., 2021).

These concepts have already gained relevance in broader mental health research, particularly in relation to common comorbidities of EDs. A growing body of evidence highlights the positive effects of probiotics, prebiotics, and dietary interventions in psychiatric conditions. For instance, individuals with major depressive disorder (MDD) and generalized anxiety disorder (GAD) have shown additional benefits when probiotics or symbiotics are used alongside standard treatments (Forth et al., 2023; Huang et al., 2016; Liu et al., 2019; Schaub et al., 2022; Sivamaruthi et al., 2019). Even more promising are emerging metagenomic insights: the presence of sporulation genes in the gut microbiota at baseline has been linked to higher chances of remission in patients treated with selective serotonin reuptake inhibitors (SSRIs) (Borgiani et al., 2025). These findings point toward the possibility of using specific gut microbiota signatures, particularly at the microbiome (functional and metagenomic) level, as biomarkers to predict antidepressant efficacy (Wang et al., 2023). Considering the high prevalence of comorbid MDD and GAD in individuals with EDs, exploring how gut microbiota may influence both eating behaviors and response to SSRIs could be helpful in developing more personalized and effective treatments (Godart et al., 2002, 2007; Swinbourne et al., 2012).

This review comes with some strengths and limitations. Its strength is that it is the first review that maps the previous scientific literature on BN/BED and gut microbiota. The inclusion of both human and murine studies contributes to a more complete understanding of the relationship between dysregulated eating behaviors and gut dysbiosis. Moreover, all results in the included study were systematically analyzed according to individual disorder categories, thereby preventing the inclusion of mixed samples that encompass AN. It is noteworthy that this approach was upheld for all studies except for two qualitative inquiries conducted by Rantala et al. (2019) and Terry et al. (2022) (Rantala et al., 2019; Terry et al., 2022). Nevertheless, the relatively limited number of identified articles, coupled with the diverse methodologies employed, introduces potential variability in the synthesized findings. Additionally, the relationship between genetic, environmental, and microbial factors in BN and BED calls for further exploration, and the existing body of literature might not capture the full range of these interactions. Finally, the current studies have not established any causal mechanisms but rather associations. To overcome these limitations, prospective research studies should incorporate larger datasets employing standardized methodologies and prioritize longitudinal designs.

In conclusion, this scoping review takes a closer look at what we currently know about how gut bacteria relate to BN and BED. Research suggests potential etiological links involving autoimmunity and PNEI mechanisms. Clinical studies have indicated that promising probiotic interventions, especially post-bariatric surgery, may mitigate eating-related outcomes. Murine models have revealed that microbiota-based interventions, such as probiotics and specific bacterial supplementation, have been shown to reduce compulsive overeating behaviors and anxiety-like behaviors in EDs, with findings highlighting sex-specific responses in these therapeutic approaches. Taken together, these findings reinforce the importance of further interdisciplinary research to better understand the gut microbiota's role in BN and BED and inform the development of targeted and effective interventions. It remains unclear what causes these patterns and future research should aim to clarify causative factors, validate potential biomarkers, and explore targeted therapeutic interventions to improve our understanding and management of these disorders.

## Informed consent statement

Not applicable.

## Institutional review board statement

Not applicable.

## Data availability statement

Not applicable.

## Author contributions

Conceptualization, S.T.V., A.R.A.; methodology, S.T.V., D.R.G., and G.G.; data curation, S.T.V., F.M., D.R.G., and G.G.; writing-original draft preparation, S.T.V., G.F., and F.M.; writing-review and editing, A.R.A. and G.F.; supervision, D.D.R. All authors have read and agreed to the published version of the manuscript.

## Funding

The research received no external funding.

## Conflicts of interest

The authors declare no conflict of interest.
