# Supplementary material for: The gut microbiota's role in bulimia nervosa and binge eating disorder: etiological insights and therapeutic implications from a scoping review
Source: Neurosci Appl. 2025 Aug 19;4:105526. doi: 10.1016/j.nsa.2025.105526 (PMC12406272; doi:10.1016/j.nsa.2025.105526)
Supplement: Multimedia component 1 [file mmc1.docx]

**Supplementary Materials**

***Pubmed Query***

((bulimia nervosa) OR (bulimia) OR (bulimic) OR (binge eating disorder)) AND ((microbiome) OR (microbiota) OR (flora) OR (microbial))

***Web of Science Query***

(("bulimia nervosa" ) OR (bulimia ) OR (bulimic ) OR ("binge eating disorder" )) AND ((microbiome ) OR (microbiota ) OR (flora ) OR (microbial ))
